# Clomiphene citrate plus letrozole versus clomiphene citrate alone for ovulation induction in infertile women with ovulatory dysfunction: a randomized controlled trial

**DOI:** 10.1186/s12905-023-02773-7

**Published:** 2023-11-14

**Authors:** Pattraporn Chera-aree, Sirikul Tanpong, Isarin Thanaboonyawat, Pitak Laokirkkiat

**Affiliations:** Infertility and Reproductive Biology Unit, Department of Obstetrics and Gynecology, Faculty of Medicine Siriraj Hospital, Mahidol University, 2 Wanglang Road, Bangkoknoi, Bangkok, 10700 Thailand

**Keywords:** Anovulation, Clomiphene citrate, Letrozole, Ovulation induction, Polycystic ovary syndrome

## Abstract

**Background:**

The aim of this study was to compare the efficacy of the combination of clomiphene citrate (CC) and letrozole to that of CC alone in inducing ovulation in infertile women with ovulatory dysfunction.

**Methods:**

A randomized controlled trial was conducted at a single academic medical center between November 2020 and December 2021. Anovulatory infertility females, aged 18 to 40, were evenly distributed by a computer-generated block of four into two treatment groups. A “combination group” received a daily dose of CC (50 mg) and letrozole (2.5 mg), while a “CC-alone group” received a daily dose of CC alone (50 mg). The study medications were administered on days 3 through 7 of menstrual cycle. The primary outcome was the ovulation rate, defined by serum progesterone levels exceeding 3 ng/mL at the mid-luteal phase. The secondary outcomes were ovulation induction cycle characteristics, endometrial thickness, conception rate, and adverse events.

**Results:**

One hundred women (50 per group) were enrolled in the study. The mean age was not significantly different in both groups: 31.8 years in the combination group and 32.4 years in the CC-alone groups (*P* = 0.54). The prevalence of polycystic ovary syndrome in the combination and CC-alone groups was 48% and 44%, respectively (*P* = 0.841). According to intention-to-treat analysis, the ovulation rates were 78% and 70% in the combination and CC-alone groups, respectively (*P* > 0.05). There was no significant difference in the mean endometrial thickness or the number of dominant follicles of the groups. No serious adverse events were observed in either group.

**Conclusions:**

Our study found no significant difference between the combination of CC and letrozole and CC alone in inducing ovulation in infertile women with ovulatory dysfunction in one cycle. The small number of live births precluded any meaningful statistical analysis. Further studies are needed to validate and extend our findings beyond the scope of the current study.

**Trial registration:**

The study was registered at https://www.thaiclinicaltrials.org with the following number: TCTR20201108004 and was approved on 08/11/2020.

**Supplementary Information:**

The online version contains supplementary material available at 10.1186/s12905-023-02773-7.

## Background

Infertility is defined as the inability to conceive through regular intercourse without contraception for 12 months in women under 35 and 6 months in women 35 or older [1]. It affects 8–12% of women globally and 12% of Thai women [2, 3]. Female-related factors, male-related factors, or both can cause infertility. Female infertility is commonly attributed to anovulation, accounting for 50% of cases [4, 5]. Hypothalamic-pituitary-ovarian dysfunction is the predominant cause of anovulation, responsible for 85% of cases, whereas polycystic ovary syndrome (PCOS) accounts for most cases of ovulatory dysfunction (30–40%) [6].

Medical induction of ovulation is a primary treatment for anovulation, particularly in patients with PCOS. Clomiphene citrate (CC), a selective estrogen receptor modulator, is commonly used for this condition [7–9]. Its mechanism of action involves competitive attachment to nuclear estrogen receptors, leading to negative feedback on estrogen. In turn, follicle-stimulating hormone is released, stimulating follicle growth and maturation. However, CC also has an antiestrogenic effect on the endometrium and cervical mucus. Prior studies reported that CC led to thinning of the endometrium and thickening of cervical mucus, adversely affecting conception despite high ovulation rates [10–13].

Letrozole has been proposed as a first-line treatment for ovulation induction in PCOS [9, 14]. The mechanism of action of letrozole is different from that of CC. Letrozole is a highly selective aromatase inhibitor that prevents androgen-to-estrogen conversion in peripheral tissues. The reduced estrogen level increases gonadotropin-releasing hormone secretion from the hypothalamus, leading to follicle growth. Unlike CC, letrozole does not affect peripheral or central estrogen receptors. Therefore, it does not lead to thinning of the endometrium, which can impair implantation. Additionally, letrozole increases follicular sensitivity to follicle-stimulating hormone due to increased androgen in the ovaries, resulting in improved follicular growth [15].

Several studies have compared the effects of CC and letrozole on infertility, particularly their impact on ovulation induction. The Pregnancy and Polycystic Ovary Syndrome II trial found a significantly higher live birth rate (27.5% vs. 19.1%; *P* = 0.007) and cumulative ovulation rate (61.7% vs. 48.3%; *P <* 0.001) in the letrozole group than in the CC group [16]. Due to the different mechanisms of action of CC and letrozole, it is possible that taking these drugs together may increase the ovulation rate by having a synergistic effect with their own mechanisms. However, few studies have compared the combination of CC and letrozole with a single ovulation induction agent. A study that evaluated the combination of CC and letrozole versus letrozole alone for ovulation induction found that the ovulation rate was significantly higher in the combination group than in the letrozole-alone group (77% vs. 42.9%, respectively; *P* = 0.007). This result suggests that CC enhances the effectiveness of letrozole for ovulation induction [10]. Hence, the potential synergistic effect of CC in a combination group should be explored. Our study aimed to compare the efficacy of CC plus letrozole with that of CC alone for ovulation induction in infertile women with ovulatory dysfunction to test the hypothesis of the additional effect of CC and letrozole.

## Methods

### Study design and overview

This study employed a randomized controlled trial design. It was conducted at the Infertility and Reproductive Biology Unit of the Department of Obstetrics and Gynecology, Faculty of Medicine Siriraj Hospital, Mahidol University, Bangkok, Thailand, from November 2020 to December 2021. Eligible participants were randomly allocated to a “combination group” or a “CC-alone group” after Institutional Review Board approval. Those assigned to the combination group received a daily dose of CC (50 mg) and letrozole (2.5 mg), whereas participants in the CC-alone group were administered a daily dose of CC alone (50 mg) [17]. Treatment medications were administered on days 3 through 7 of each participant’s menstrual cycle. The study enrolled infertile Thai women aged between 18 and 40 with ovulatory dysfunction (cycle length > 35 days or diagnosed with PCOS according to the modified Rotterdam criteria [18–20]). Participants were excluded if they had spontaneous pregnancy, uncorrected thyroid disease, hyperprolactinemia, allergy or contraindication to letrozole or CC, bilateral tubal occlusion, or a male partner with a total motile sperm count less than 10 × 10^6^ [21]. Before this research began, the Siriraj Institutional Review Board approved its protocol (Si-257/2020), and all participants provided written informed consent to participate. The study was registered at https://www.thaiclinicaltrials.org (TCTR20201108004) on 08/11/2020 and followed the CONSORT and IMPRINT guidelines [22]. The overview and timeline of the method are shown in Supplementary Fig. 1.

### Randomization and blinding

The randomization scheme in this study was computer-generated using blocks of four, with group assignments concealed in sealed envelopes. The sonographer was blinded to the assignments. Participants were randomized in a 1:1 ratio to receive a daily dosage of CC (50 mg; Ovamit, Remedica Ltd, Limassol, Cyprus) in combination with letrozole (2.5 mg; Fresenius Kabi Oncology Ltd, Kolkata, India) or CC alone (50 mg) daily. Both treatment regimens were taken from days 3 through 7 of the menstrual cycle. Patients with long menstrual cycles were prescribed an oral progestogen to induce withdrawal bleeding. The progestogens administered were medroxyprogesterone acetate (10 mg daily; Provera; Pfizer, New York, NY, USA) or norethisterone acetate (5 mg daily; Primolut N; Bayer Thai Co Ltd, Bangkok, Thailand) for 7–10 days.

### Study procedures

At the start of their menstrual cycle, participants were instructed to contact the investigator to arrange the ovulation induction schedule. The allocated treatment medication regimen was taken from days 3 through 7 of one menstrual cycle. Home urinary luteinizing hormone (LH) tests were performed twice daily, in the morning and at night, starting on cycle day 12 until a positive result was obtained or until cycle day 21 if the results continued to be negative. Patients were instructed to send pictures of the urinary LH test results to the researcher to confirm the results. Regular intercourse, performed two to three times per week, was recommended starting on cycle day 12 and on the day of the positive urinary LH test. Transvaginal ultrasound was performed on day 12 to day 14 of the cycle by a single operator throughout the project, and follicular growth and endometrial thickness were recorded. Serum progesterone levels were obtained 7 days after a positive urinary LH test or on cycle day 21 or day 22 in cases with negative urinary LH. A urine pregnancy test was performed 7 days after an ovulatory serum progesterone level or on cycle day 35 if there was no confirmation of ovulation and no menstrual period. Women with a positive urine pregnancy test were scheduled for a transvaginal ultrasound to confirm pregnancy 2 to 3 weeks later. A questionnaire was used to elicit information about any adverse effects of the study medications during the study period.

### Outcome measures

The primary outcome was the ovulation rate, defined as a mid-luteal progesterone level greater than 3 ng/mL [23]. The secondary outcomes were the following: medically related side effects, including headache, dizziness, and hot flush, etc.; pregnancy complications or congenital anomalies; ovulation induction cycle characteristics, including endometrial thickness and the number of preovulatory mature follicles (defined as follicles with diameters ≥ 14 mm [24]); conception rate (diagnosed by a positive urine pregnancy test); clinical pregnancy rate (confirmed by positive fetal heartbeat on transvaginal ultrasonography); and live birth.

### Sample size calculation and statistical analyses

The sample size calculation was informed by Meija et al. [10] and another study [16]. Meija et al. reported a 77% ovulation induction rate in women with PCOS treated with a combination of CC and letrozole. In contrast, the second study found a 48% rate for those treated with CC alone. A power analysis was conducted to determine the number of participants needed to detect a clinically meaningful difference in the ovulation rates of the 2 study groups. We calculated that 43 subjects per group would be required to achieve 80% statistical power (β) with a two-sided significance level (α) of 0.05. The sample size was increased to 50 participants per group to account for potential dropouts.

The statistical analyses were performed using PASW Statistics for Windows, version 18.0 (SPSS Inc, Chicago, IL, USA). The study conducted an intention-to-treat analysis involving all randomized participants and a per-protocol analysis restricted to those who followed the designated treatment. Descriptive statistics were used to describe patient and cycle characteristics. Continuous data were presented as the means ± standard deviations or medians (ranges) for normally distributed and nonnormally distributed data, respectively, whereas categorical data were expressed as numbers and percentages. For categorical data, Pearson chi-squared, Yates’ continuity correction, or Fisher’s exact test were performed to compare the proportions between two groups. The independent Student’s t-test was used for normally distributed continuous data, while the Mann-Whitney U-test was used for nonnormally distributed continuous data for comparing the mean or median, respectively, between two groups. Absolute differences were computed by substracting percentages of the combination group’s outcomes from percentages of the CC-alone group’s outcomes, while the rate ratio was computed by percentages of the combination group’s outcomes divided by percentages of the CC-alone group’s outcomes. The level of statistical significance was set at *P* < 0.05 for all tests.

## Results

**Fig. 1:**
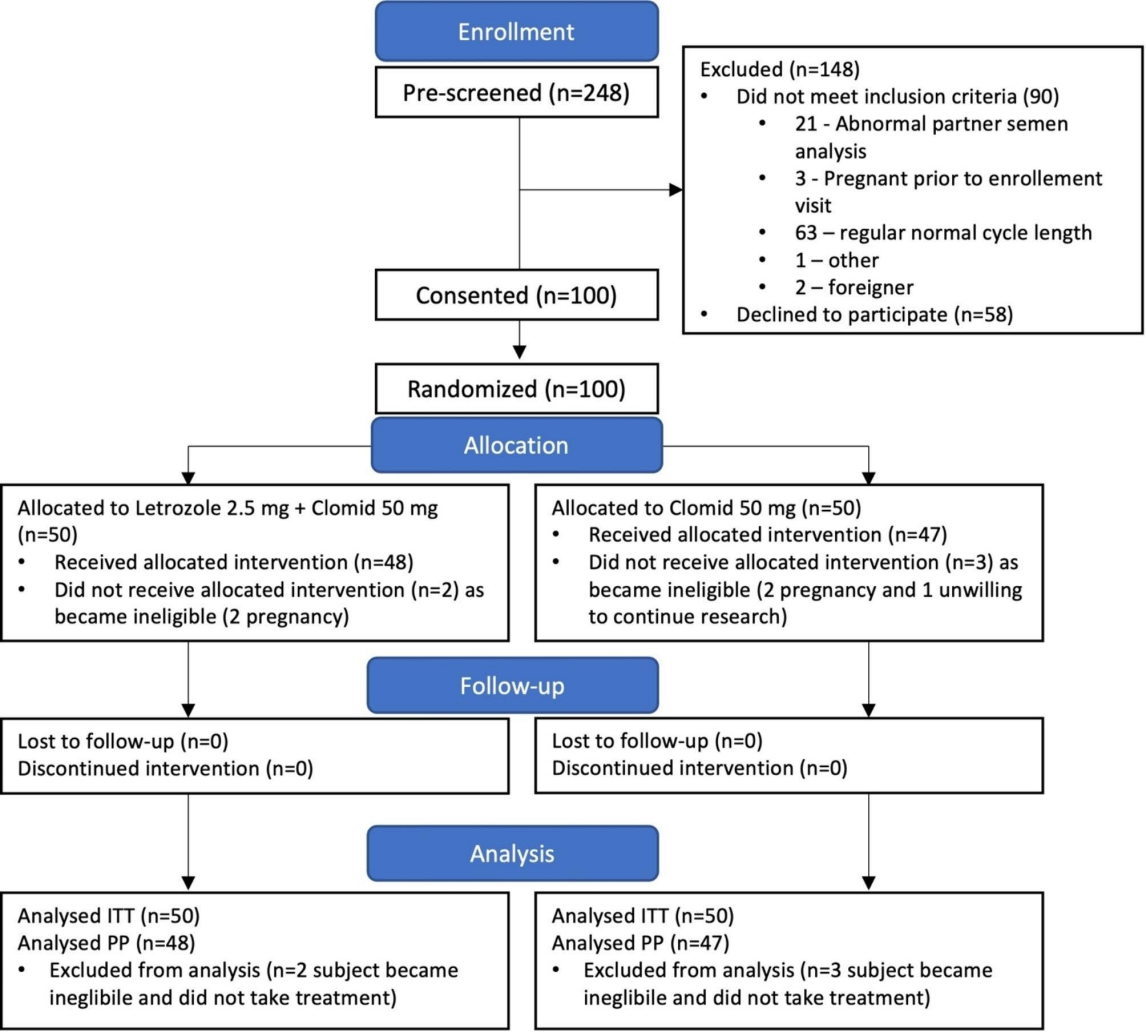
Flow diagram of the study protocol

After enrolling 248 patients, there were one hundred women with ovulatory dysfunction who were equally randomized into either the combination group or the CC-alone group. Before starting treatment, five women were excluded (four due to spontaneous pregnancy, and one decided not to continue with the study). Ninety-five participants received the allocated treatments (48 in the combination group and 47 in the CC-alone group; Fig. 1). The baseline characteristics of the participants are listed in Table 1. There were no significant differences between the groups concerning mean age, mean body mass index, or baseline total motile sperm counts. The combination group had a mean age of 31.8 ± 4.6 years, while the CC-alone group had a mean age of 32.4 ± 3.8 years. The mean body mass indexes for both groups were in the normal range: combination group, 23.5 ± 4.9, and CC-alone group, 22.9 ± 3.5 kg/m2. The baseline total motile sperm counts were also comparable, with 48.7 million for the combination group and 55.3 million for the CC-alone group. The median duration of attempting to conceive was 2 years in both groups. According to the modified Rotterdam criteria, the prevalence of PCOS in the groups was also comparable (48% in the combination group and 44% in the CC-alone group; *P* = 0.841).

**Table 1:** Comparison of the baseline characteristics of the CC + letrozole and CC-alone groups

Characteristic	Letrozole + CC group (n = 50)	Clomiphene citrate group (n = 50)	P-values
Age (years), mean ± SD	31.8 ± 4.6	32.4 ± 3.8	0.540
BMI (kg/m^2^), mean ± SD	23.5 ± 4.9	22.9 ± 3.5	0.456
Fertility history			
Previous live birth, n (%)	5 (10.0%)	2 (4.0%)	0.436
Previous abortion, n (%)	13 (26.0%)	7 (14.0%)	0.211
Duration of attempts to conceive (years), median (min, max)	2 (1, 11)	2 (1, 10)	0.554
Ovulatory disorders			
PCOS	24 (48.0%)	22 (44.0%)	0.841
Unexplained ovulatory disorders	26 (52.0%)	28 (46%)	
Coexisting condition, n (%)			
Endometriosis	5 (10.0%)	12 (24.0%)	0.110
Myoma uteri^+^	5 (10.0%)	5 (10.0%)	1.000
Baseline total motile sperm count (millions), median (min, max)	48.7 (10, 418)	55.3 (10, 350)	0.915

**PCOS, polycystic ovary syndrome**

^**+**^ Submucous or intramural myoma, distorted the uterine cavity

- None of the patients in either group had other coexisting conditions (pelvic inflammatory disease, autoimmune disease, and recurrent pregnancy loss)

The reproductive outcomes of the study participants are presented in Table 2. The ovulation rate in the combination group was slightly higher than that in the CC group, but the difference was not statistically significant. In the intention-to-treat analysis, the ovulation rate was 78% (39 out of 50) in the combination group compared to 70% (35 out of 50) in the CC group (*P* = 0.494). In the per-protocol analysis, the ovulation rate was 77% (37 out of 48) in the combination group and 70% (33 out of 47) in the CC group (*P* = 0.447). Five participants conceived after completing the ovulation induction cycle (three from the combination group and two from the CC group). Among these, 2 pregnancies in the combination group ended in first-trimester pregnancy loss, and one resulted in a clinical pregnancy that continued to live birth. In the CC group, 1 participant experienced first-trimester pregnancy loss, and another continued to live birth.

**Table 2:** Comparison of the reproductive outcomes of the CC + letrozole and CC-alone groups

Outcome	Letrozole + CC	Clomiphene citrate	Absolute difference between groups (95% CI)^a^	Rate ratio in combination group (95% CI)^b^	P-values
**Primary outcome**					
Ovulation, n (%)					
Intention to treat analysis^†^	39 (78)	35 (70)	8.0 (-9.1 to 24.6)	1.11 (0.88 to 1.41)	0.494
Per-protocol analysis^‡^	37 (77)	33 (70)	6.9 (-10.7 to 24.0)	1.10 (0.86 to 1.40)	0.447
**Secondary outcomes**					
Pregnancy, n(%)					
Conception	3 (6.3)	2 (4.3)	2.0 (-8.8 to 13.0)	1.47 (0.26 to 8.40)	1.000
Clinical pregnancy	1	2	NA*	NA*	NA*
Live birth	1	1	NA*	NA*	NA*
Pregnancy loss	2	1	NA*	NA*	NA*
Fecundity among those who ovulated					
Conception	3/37	2/33	2.0 (-12.5 to 15.9)	1.34 (0.24 to 7.52)	1.000
Clinical pregnancy	1/37	2/33	NA*	NA*	NA*
Live birth	1//37	1/33	NA*	NA*	NA*

^†^ Letrozole + CC, n = 50; CC, n = 50

^‡^ Letrozole + CC, n = 48; CC, n = 47

^a^ Differences are presented as percentages

^b^ Differences are presented as ratio

NA* not applicable due to small sample

Ovulation induction cycle characteristics are detailed in Table 3. The proportion of participants who experienced progestin-induced withdrawal bleeding was 35.4% in the combination group and 29.8% in the CC group (*P* = 0.714). Transvaginal sonography was performed on median day 13 of the menstrual cycle in both groups. The positive urine LH surge rate was non-significantly lower in the combination group than in the CC group (60.4% vs. 72%; *P* = 0.311). The median number of women with follicles > 14 mm and the median size of the largest follicle in the combination group and CC group were comparable (64.6% vs. 63.8%, with *P* = 1.000; and 16.75 mm vs. 15.5 mm, with *P* = 0.687, respectively). The mean endometrial thickness was 6.85 mm in the combination group and 7.40 mm in the CC group (*P* = 0.171). The ovulation rates in PCOS patients receiving combination drugs versus the CC alone group were 60.9% (14 out of 23) and 60% (12 out of 20), respectively (*P* = 1.000). Among participants who ovulated, the median number and size of dominant follicles and endometrial thickness of the groups were also comparable.

**Table 3:** Comparison of the cycle characteristics of the CC + letrozole and CC-alone groups

Characteristic	Letrozole + CC group (n = 48)	Clomiphene citrate group (n = 47)	P-values
Progestin withdrawal, n (%)	13 (35.4)	14 (29.8)	0.714
Ultrasound cycle day, median (min, max)	13 (11, 15)	13 (12, 16)	0.940
Reported urine LH surge, n (%)	29 (60.4)	34 (72)	0.311
Cycle day of LH surge median (min, max)	(n = 29) 16 (12, 21)	(n = 34) 15 (12, 18)	0.191
No. of follicles > 10 mm, median (min, max)	1 (0, 5)	1 (0, 5)	0.815
No. of follicles > 14 mm, median (min, max)	1 (0, 5)	1 (0, 5)	0.919
No. of women with follicle > 14 mm, n (%)	31 (64.6)	30 (63.8)	1.000
Largest follicle size (mm), median (min, max)	16.75 (0, 35)	15.50 (5.5, 35.5)	0.687
Endometrial lining thickness (mm), median (min, max)	6.85 (3.1, 13.5)	7.40 (3.6, 12.6)	0.171
Cycle day progesterone level obtained, median (min, max)	22 (19, 28)	22 (19, 28)	0.768
Progesterone level, ng/ml, median (min, max)	18.40 (0.05, 60)	17.90 (0.05, 60)	0.387
**Cycle characteristics of those who ovulated**	**Letrozole + CC group** **(n = 37)**	**Clomiphene citrate group** **(n = 33)**	
Progestin withdrawal, n (%)	10 (27)	9 (27.3)	1.000
Ultrasound cycle day, median (min, max)	13 (11, 15)	13 (12, 15)	0.197
Reported urine LH surge, n (%)	28 (75.7)	28 (84.8)	0.510
Cycle day of LH surge, median (min, max)	16 (12, 21)	15 (12, 18)	0.379
No. of follicles > 10 mm, median (min, max)	2 (0, 5)	2 (0, 5)	0.922
No. of follicles > 14 mm, median (min, max)	1 (0, 5)	1 (0, 5)	0.712
No. of women with follicles > 14 mm, n (%)	29 (78.4)	27 (81.8)	0.952
Largest follicle size, mm, median (min, max)	17.6 (0, 35)	20.5 (9.5, 35.5)	0.572
Endometrial lining thickness, mm, median (min, max)	7 (3.5, 13.5)	8 (3.6, 12.6)	0.441
Cycle day progesterone level obtained, median (min, max)	22 (19, 28)	22 (19, 28)	0.990
Progesterone level, ng/ml, median (min, max)	27.80 (3.5, 60)	25.10 (3.28, 60)	0.860

Drug tolerability and adverse events are presented in Table 4. No anaphylaxis related to treatment occurred during this study. All reported adverse events were minor and tolerable, and all participants who reported side effects were willing to continue their allocated medication. There were no significant differences in the side effect profiles of the groups, with abdominal bloating being the most common side effect in both groups. There were no congenital defects in any of the live births.

**Table 4:** Drug tolerability and adverse events compared between the CC + letrozole and CC alone groups

Event	Letrozole + CC group (n = 48)	Clomiphene citrate group (n = 47)	P-values
Anaphylaxis, n	0	0	–
Minor side effects, n (%)	24 (50)	22 (46.8)	0.916
Side-effects acceptable	24 (100)	22 (100)	–
Reported side-effects, n (%)			
Headache	3 (12.5)	1 (4.5)	0.609
Dizziness	2 (8.3)	4 (18.2)	0.405
Hot flush	5 (20.8)	1 (4.5)	0.190
Abdominal bloating	7 (29.2)	6 (27.3)	1.000
Abdominal pain including cramping	3 (12.5)	2 (9.1)	1.000
Nausea	2 (8.3)	1 (4.5)	1.000
Mood changes	4 (16.7)	3 (13.6)	1.000
Fatigue	5 (20.8)	3 (13.6)	0.702
Breast discomfort	5 (20.8)	5 (22.7)	1.000
Diarrhea	5 (20.8)	2 (9.1)	0.418
Night sweats	4 (16.7)	1 (4.5)	0.349
Sleep disturbance	3 (12.5)	5 (22.7)	0.451
Adverse events of ongoing pregnancy, n (%)			
Spontaneous complete abortion	1 (2.08)	1 (2.13)	NA*
Early pregnancy loss	1 (2.08)	1 (2.13)	NA*

NA* not applicable due to small sample

## Discussion

This study aimed to compare the efficacy of a combination of CC and letrozole versus CC alone for ovulation induction in infertile women with ovulatory dysfunction. A previous study reported that women with PCOS had a significantly higher ovulation rate with combination therapy than with letrozole alone [10]. This better outcome may have been due to complementary action between the 2 agents. However, the causal relationship between combination therapy and increased ovulation and pregnancy rates remains unclear. Therefore, our study was designed to compare the efficacy of the combination therapy and CC alone to investigate whether our results could support the hypothesis. Interestingly, our investigation found no significant difference in the ovulation rates of our 2 study groups.

From a theoretical standpoint, the local action of letrozole and the central influence of CC were anticipated to synergistically enhance ovulation induction. However, our study found comparable ovulation rates for the combination and CC-alone groups. Furthermore, the dose of CC was identical in both groups of our study, and a previous study reported a higher ovulation rate in a combination group than in a letrozole-alone group [10]. These results suggest that CC is the primary driver of ovulation.

Numerous studies have investigated the individual effects of letrozole and CC on the ovulation rate; however, studies examining the combined effect of these medications are limited. A study by Hajishafiha et al. investigated the combination of letrozole and CC in PCOS patients and reported a follicle development rate of 82.9% in the combination group. However, that study included patients who were refractory to CC or letrozole, and ovulation confirmation using follicle size alone (as was done by Hajishafiha et al.) is not always reliable [11]. Another randomized controlled trial compared the combination of letrozole and CC with letrozole alone for ovulation induction in women with naive PCOS and reported an ovulation rate of 77% in the combination group [10]. Although that study introduced an interesting concept (combining CC and letrozole for ovulation induction), it had a small sample size and lacked a comparison with a CC-alone group. Additionally, the participants in the 2 previous studies exhibited heterogeneity compared with the current investigation. Furthermore, the primary outcome of the work by Hajishafiha et al. differed from those of the randomized controlled trial and our investigation. Our data show no significant difference in the ovulation rates of the CC plus letrozole and CC-alone groups. This finding implies that CC alone may serve as an economically viable ovulation induction agent for both generalized anovulatory women and non-CC-resistant PCOS women.

Previous meta-analyses have reported significant heterogeneity among randomized controlled trials investigating the ovulation rates achieved with letrozole and CC in the PCOS population, although letrozole significantly increased the ovulation rate compared to CC [25, 26]. In contrast, a meta-analysis of studies on unexplained infertility in women that compared the efficacy of letrozole with that of CC found no significant differences in the clinical outcomes of the 2 groups. There was also significant heterogeneity across the included studies [27]. Interestingly, our study showed higher ovulation rates in both treatment groups than those reported by a study using CC and letrozole alone [10, 28]. However, the ovulation rates of our study’s 2 treatment groups were similar to that of the combination group in work by Mejia et al. [10, 28] Differences in study populations may explain these discrepancies. We included both PCOS and generalized anovulatory patients, the latter of whom may respond better to ovulation induction agents, and all of our participants were naive to ovulation induction.

The side effects of the 2 groups in our study were comparable, and all adverse effects from medication were minor and tolerable. Abdominal bloating was the most common side effect reported in both groups. We also found no evidence of congenital anomalies in the live births from either group. Taken together, these results indicate that both treatment regimens were safe and well tolerated.

The observations made in this study represent a significant contribution to the literature. Notably, our study is the first to compare the efficacy of ovulation induction achieved with the combination of CC and letrozole versus CC alone in anovulatory women. Our study’s randomized controlled trial design minimized confounding factors and neutralized baseline characteristics between groups. Additionally, mid-luteal serum progesterone clearly defined the primary outcome, which was unaffected by operator and participant biases. To minimize interobserver variation, a single operator blinded to the study medications performed all transvaginal ultrasonographic investigations.

Several limitations should be acknowledged in this study. First, the low pregnancy rate may limit the generalizability of our findings, as we did not evaluate all possible infertility factors that could have contributed to the low rate. Second, while we advised participants on the optimal timing of intercourse, we could not determine the degree of adherence to these instructions. Third, our study’s treatment and follow-up durations were relatively short, as we evaluated only one treatment cycle in each patient. Finally, the letrozole-only group for ovulation induction was not included in this study. Therefore, more treatment groups and long follow-up periods are needed to assess and compare the cumulative ovulatory rate between these ovulation induction regimens. As we know that live birth is the most meaningful outcome, the ovulation rate was a reasonable primary outcome for evaluating the efficacy of ovulation induction agents. This is because the rate minimizes confounding factors related to infertility factors beyond ovulation.

## Conclusions

Our study found no significant difference in the ovulation rates of infertile women with ovulatory dysfunction. Specifically, the rates achieved with a combination of CC and letrozole were not significantly different from those achieved with CC 50 mg alone in one cycle. However, the low number of live births precluded statistical analysis. According to the limitations mentioned above, future studies with larger sample sizes and extended follow-up periods or equivalence trials are needed to evaluate the cumulative ovulatory rates and dose-defining efficacy of these ovulation-induction regimens.

### Electronic supplementary material

Below is the link to the electronic supplementary material.

Supplementary Material 1

## Data Availability

The datasets used and/or analyzed during the current study are not publicly available due to the confidentiality of participants’ data and the difficulty of organizing the raw data to be suitable for publication; however, they are available from the corresponding author on reasonable request.
