# Supplementary figures and images for: Clomiphene citrate plus letrozole versus clomiphene citrate alone for ovulation induction in infertile women with ovulatory dysfunction: a randomized controlled trial
Source: BMC Womens Health. 2023 Nov 14;23:602. doi: 10.1186/s12905-023-02773-7 (PMC10647029; doi:10.1186/s12905-023-02773-7)

**
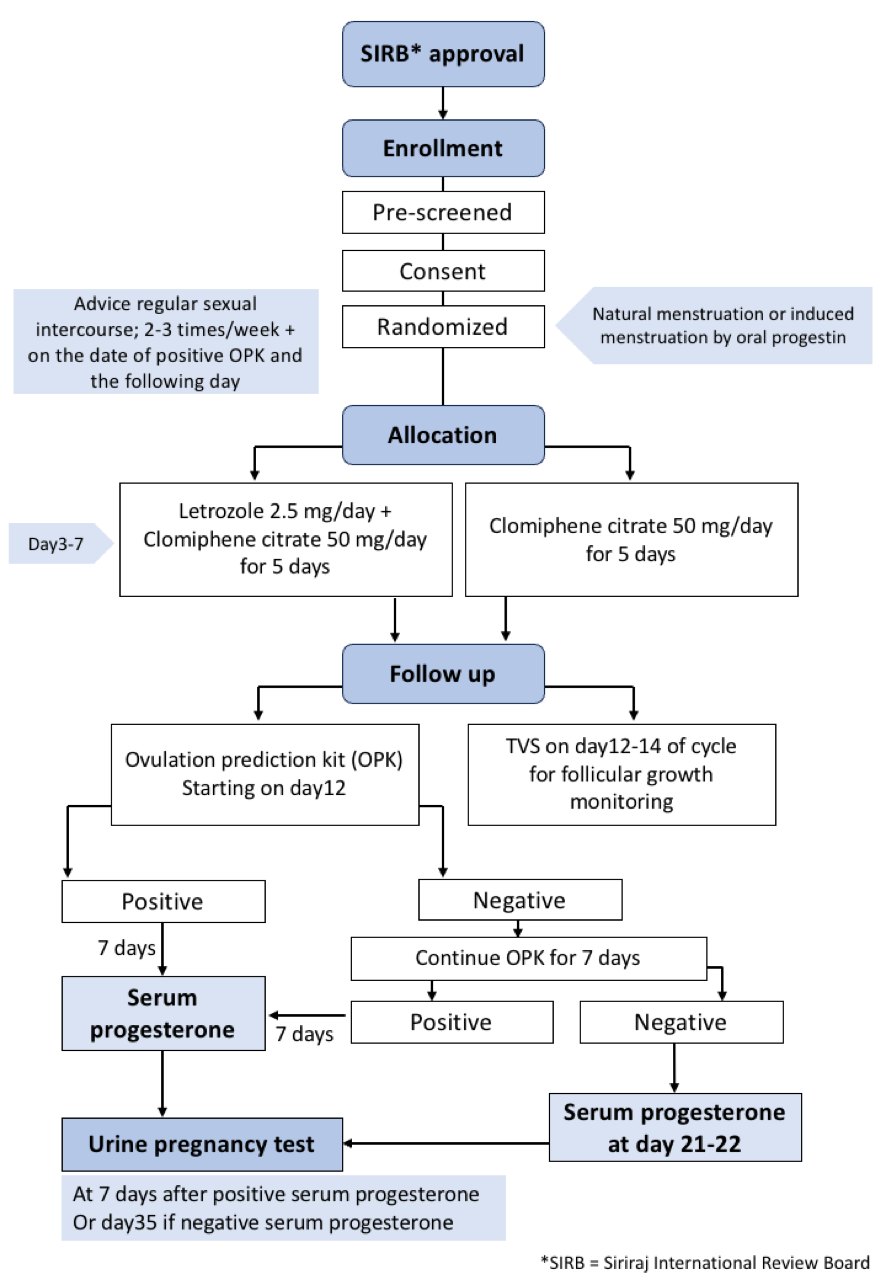
Supplementary Figure1.** The overview and timeline of the study method

Supplement: Supplementary file 1 — Supplementary Material 1 [file 12905_2023_2773_MOESM1_ESM.docx]
